# Relationship between individual ventilatory threshold and maximal fat oxidation (MFO) over different obesity classes in women

**DOI:** 10.1371/journal.pone.0215307

**Published:** 2019-04-11

**Authors:** Gian Pietro Emerenziani, Dafne Ferrari, Chiara Marocco, Emanuela A. Greco, Silvia Migliaccio, Andrea Lenzi, Carlo Baldari, Laura Guidetti

**Affiliations:** 1 Department of Experimental and Clinical Medicine, University of Magna Graecia of Catanzaro, Catanzaro, Italy; 2 Department of Movement, Human and Health Sciences, Section of Health Sciences, University of Rome “Foro Italico”, Rome, Italy; 3 Department of Experimental Medicine, Section of Medical Pathophysiology, Endocrinology and Nutrition, Sapienza University of Rome, Rome, Italy; 4 University eCampus, Novedrate, Italy; University of Bourgogne France Comté, FRANCE

## Abstract

**Objective:**

The use of the Individual Ventilatory Threshold (IVT), as parameter to prescribe exercise intensity in individuals with obesity, has become more frequent during the last years. This study aimed to evaluate the relationship between IVT and Maximal Fat Oxidation (MFO) in women with obesity.

**Methods:**

Fifty-two obese female adults (age = 43.6±10.9 years; BMI = 38.5±5.2 kg/m^2^) were included in this study. According to the BMI classification, subjects were divided into three groups: Obese Class I (OBI, n = 16); Obese Class II (OBII, n = 20) and Obese Class III (OBIII, n = 16). All subjects performed an incremental graded exercise test to evaluate peak oxygen uptake (VO_2peak_), IVT and MFO. MFO was evaluated using a stoichiometric equation. Fat max zone was determined for each subject within 10% of fat oxidation rates at MFO. For each HR, %HR_max_, VO_2_ and %VO_2peak_ variable, Pearson’s correlation test was done between IVT and MFO exercise intensity. When statistical correlation was found we used a comparative statistical analysis to assess differences between IVT and MFO. Statistical significance was set at P ≤ 0.05.

**Results:**

For each HR, %HR_max_, VO_2_ and %VO_2peak_ variable there was a positive significant correlation (P<0.01) between IVT and MFO. No significant differences were found for HR, %HR_max_, and VO_2_ between IVT and MFO. %VO_2peak_ was significantly higher at IVT than at MFO (P = 0.03).

MFO rates were significantly higher in OBIII women than in women of the other two classes. In all subjects, IVT was within the fat max zone.

**Conclusion:**

The use of HR and VO_2_ corresponding to IVT could be a useful parameter not only to improve cardiorespiratory fitness but also to prescribe physical activity that maximize fat oxidation in obese subjects.

## Introduction

Obesity is a condition characterized by an increased fat accumulation that impairs health and, according to the World Health Organization (WHO) over 650 million of subjects were obese in 2016 [1]. One of the best approaches to contrast the development of obesity is an healthy lifestyle which should combine physical activity (PA) with a balanced diet. According to the WHO classification, there are three different classes of obesity based on BMI values: obesity Class I (OBI) when BMI is between 30–34.9 kg/m^2^, Obese Class II (OBII) when BMI is between 35–39.9 kg/m^2^ and Obese Class III (OBIII) when BMI is ≥ 40 kg/m^2^.

It is known that the combination of moderate PA and diet has positive effects on body composition and cardiovascular parameters in obese subjects [2, 3]. Recently, the use of Individual Ventilatory Threshold (IVT), as parameter to individualize the exercise intensity in obese population, has become more frequent [4]. Indeed, the heart rate corresponding to the IVT allows to individualize exercise intensity at which the subjects use mainly an aerobic metabolism and it could be useful as parameter to prescribe the exercise intensity according to the real subjects’ health condition [5]. Moreover, IVT might leads the exercise to be better tolerated in subjects with obesity than other exercise intensities. For instance, the use of the heart rate corresponding to the IVT in obese subjects has been demonstrated to have positive results on body composition and cardiorespiratory parameters [6, 7]. Results of our group [6] demonstrated that after 4-month of unsupervised aerobic training based on IVT, obese subjects obtained an improvement in both functional and aerobic capacity. Moreover, to realize a correct exercise program aiming to lose weight, it is very important to consider the exercise intensity that elicits maximal fat oxidation rates. Indeed, several international groups have focused their research on the evaluation and characterization of which exercise intensity may produce maximal fat oxidation (MFO) [8, 9, 10,11]. The exercise intensity at which MFO occurs is termed Fat_max_.

Fat_max_ is usually allocated at the intensity at which glycolysis is increased considerably; after that fat oxidation is inhibited by the high rates of glycolysis [12, 13]. Scientific evidence have showed that the exercise intensity at which MFO occurs (Fat_max_) ranging from 33 and 65% of subjects’ VO_2max_ [14].High intensities training may lead to a higher fat oxidation rates than low-moderate exercise intensity at fixed time length. However, obese subjects showed to have a low cardiorespiratory efficiency and a low exercise tolerance. Consequently, the use of low-moderate exercise intensity (IVT) may be more tolerable. Moreover, in obese subjects exercising at low-moderate exercise intensities has been showed to have positive results in promotion of PA [15] (e.g. increasing the compliance to the exercise program) and might be useful so that a greater number of patients could benefit from regular physical exercise in an almost daily routine [6]. Therefore, exercising at IVT plus diet might increase the negative energy balance and it could be helpful in order to lose weight and body fat. Fat_max_ zone is defined as the range of exercise intensities with fat oxidation rates within 10% of fat oxidation rates at Fat_max_ [8, 16]. Fat oxidation rate is influenced by exercise intensity; in fact, fat oxidation increases from low-to-moderate intensities before declining after MFO, while carbohydrate oxidation increases linearly with exercise intensity. Therefore, an increase in exercise intensity makes carbohydrate the predominant source. Interestingly, endurance training at Fat_max_ resulted to an increase of 44% in fat oxidation rates in obese male subjects [17]; similar results have been shown not only in obese males [18] but also in obese females [10] and in untrained subjects [19]. The effects of 8 and 10 weeks of endurance exercise training, performed at Fat_max_ intensities, and interval training at ± 20% Fat_max_ intensities, were analyzed in overweight and obese men (Class I obesity) showing that a low exercise intensity could improve fat oxidation in this population [17]. In contrast, Lanzi et al. [20] demonstrated that there were no differences in aerobic fitness (VO_2peak_) and fat oxidation rates after 2-weeks of physical activity at Fat_max_ intensity between men with class II and III obesity. A cardiopulmonary exercise test (CPET) is used to assess physiological parameters at Fat_max_ however, this practice requires qualified technical staff and sophisticated equipment [21]. Nevertheless, CPET is considered the gold-standard method to assess subjects’ cardiorespiratory parameters, we have previously [22] demonstrated that the six-minute walking test can be used as tool to estimate the HR at exercise intensity corresponding to IVT in obese subjects. Therefore, it would be very interesting to analyze the relationship between IVT and Fat_max_ because, whether there are no differences between them, the use of 6-MWT may be helpful also to estimate the exercise intensity at which maximal fat oxidation occurs. Consequently, Fat_max_ could be estimated using the practical and inexpensive 6-MWT. Accordingly, using a field test, the exercise specialist might prescribe the exercise intensity in order to increase compliance to the exercise and therefore to increase the negative energy balance. To the best of our knowledge, no previous study focused on the relationship between IVT and MFO in in women adults with obesity. Thus, the aim of this study was to describe and examine the relationship between IVT and MFO in class I, II and III obese females.

## Materials and methods

### Participants

Fifty-two obese women (age = 43.6±10.9 years; BMI 38.5 ± 5.2 kg/m^2^) were recruited in this study among individuals admitted to the Day Hospital of the Department of Experimental Medicine, Section of Medical Pathophysiology, Endocrinology & Nutrition, Policlinico Umberto I, “Sapienza University” of Rome. All participants underwent clinical examination to exclude any contraindications to PA. The inclusion criteria were adult age (18–65 years) and BMI ≥30 kg/m^2^. The exclusion criteria were neuropathy, autonomic dysfunction, cardiovascular diseases (myocardial infarction during the previous six months, myocardial ischemia or ventricular tachycardia, obstructive valvular heart disease), uncontrolled hypertension (blood pressure values exceeding 140 mm Hg systolic or 90 mm Hg diastolic), thyroid diseases including autoimmunity, or any treatment with thyroid hormone preparations or amiodarone, methimazole or propylthiouracil in the prior three months. During clinical examination all subjects reported to be sedentary and not being engaged in any organized PA.

All subjects provided a written informed consent before the beginning of the study. This study was conducted according to the Declaration of Helsinki and approved by the Sapienza University of Rome Ethical Committee (approval number 70/11, 2011).

### Experimental design

According to the WHO [23], subjects were divided into three groups based on BMI values: Obese Class I (OBI, 30<BMI<34.9 kg/m^2^, n = 16); Obese Class II (OBII, 35<BMI<39.9 kg/m^2^, n = 20), Obese Class III (OBIII, BMI≥40 kg/m^2^, n = 16). The sample included 13 subjects who were in menopause condition. Menopause condition was equally distributed among the three obesity classes groups X^2^ (2, N = 52) = 1.73, p = 0.42. Moreover, subjects did not refer to use oral contraception or to be treated with hormone replacement therapy.

After basal clinical examination, women enrolled in the study performed a series of tests to analyze body composition and physiological capacities. All tests were performed at the Department of Movement, Human and Health Sciences at the University of Rome “Foro Italico”. Cardiopulmonary exercise test (CPET) on treadmill was performed to evaluate peak oxygen uptake, IVT and MFO.

All subjects were tested in the morning (between 9 and 12 AM), and under similar environmental conditions (temperature 21–22° C; humidity 50–60%). Subjects had their usual breakfast at least one hour and half before the test session.

### Anthropometric and body composition measurements

Body weight and height were measured using a scale and a stadiometer to the nearest 0.1 kg and 0.1 cm, respectively. Body composition was measured by bioelectrical impedance method (BIA AKERN 101 Anniversary, Pontassieve, FI, Italy) while the subjects wore minimal clothing (i.e., underwear), in thermoneutral state and one hour and an half after their usual breakfast.

### Cardiorespiratory capacity

Prior to the test session, participants took part in a familiarization session (one week before the test session) to become accustomed to tests. During the test session, each subject rested quietly for 5 min to assess the resting HR. The mean HR of the last minute was taken as the resting value. A CPET on a treadmill (Woodway PRO, Woodway, Waukesha, WI, USA) was used to evaluate the peak oxygen uptake (VO_2peak_). Breath by breath measures of VO_2_, carbon dioxide production (VCO_2_), and VE were measured (Quark RMR-CPET Cosmed^™^, Rome, Italy) [24]. The system was calibrated, prior to each test, according to the manufacturer’s instructions. In detail, the two-point gas calibration was completed sampling the ambient air and the gas from a certified tank containing 16% O_2_, 5% CO_2_ and standard atmospheric Nitrogen. The CPET on treadmill protocol started at 3 km/h, then the speed was increased by 1 km/h every 3 min until 5 km/h was reached. Then, slope was increased by 3% every 3 min until subjects declared to perceive a fatigue corresponding to a value of 10 on RPE-OMNI-Walk/Run Scale [25]. The 3 min exercise protocol has been used previously to determine substrate oxidation in obese population [17]. HR (beats∙min) was continuously recorded using a HR monitor (RS 400, Polar Electro^™^, Kempele, Finland). During the test, the highest VO_2_ attained was chosen as the VO_2peak_. The individual ventilatory threshold (IVT) was determined offline for each subject by plotting the ventilatory equivalent of oxygen (VE/VO_2_) as a function of VO_2_ to identify mathematically and visually the point where this curve reached its lowest value during the exercise test [26]. The level of VO_2_ at which we observed the lowest value of the VE/VO_2_, in the individual plot, was the individual ventilatory threshold [26]. The exercise intensity corresponding to IVT was reached by all subjects. During graded exercise test all subjects reach a value of respiratory exchange ratio (RER) equal to 1.0. Subjects’ HR_max_ was calculated using the following equation: 208–0.7 × age [27]. A standard definition of OMNI-Walk/Run Scale was explained to the subjects immediately before the exercise test. During exercise testing, subjects were asked to report the degree of exertion on the OMNI-Walk/Run scale every 3 min.

### Indirect Calorimetry and Calculations

The average values for VO2 (L/min) and VCO2 (L/min), obtained during the graded exercise test, were calculated over the last 2 minutes of each stage [8].

Fat oxidation rate was calculated by using the stoichiometric equation (FAToxi (g/min) = 1.695‧VO2–1.701‧VCO2–1.77∙n) for all exercise intensities, with the assumption that urinary nitrogen excretion was negligible (n = 0) [12]. For each subject, a best-fit polynomial curve (2^nd^ order) was constructed using fat oxidation rate (expressed as g/min) vs. exercise intensity (expressed as HR and VO_2_). The peak rate of fat oxidation measured over the entire range of exercise intensities (MFO), the exercise intensity at which the MFO was observed (Fat_max_) and the range of exercise intensities with fat oxidation rates within 10% of fat oxidation rates at Fat_max_ (Fat_max_ zone), were calculated for all subjects.

### Statistical analysis

The Shapiro-Wilk test was used to ensure normally distributed data. Levene’s test was used to evaluates the homogeneity of the data. All results were expressed as mean ± SD. Differences between groups on body composition (weight, BMI, FFM, %FFM, %FM), age, and VO_2peak_ were evaluated with a one-way ANOVA. An independent t-test was conducted on overall sample to compare pre-menopause and menopause conditions in HR, %HR_max_, VO_2_, and %VO_2peak_ variables.

For each HR, %HR_max_, VO_2_ and %VO_2peak_ variable, Pearson’s correlation test was done between IVT and MFO exercise intensity. The correlation coefficient R and P value were calculated. Moreover, when statistical correlation was found we used a comparative statistical analysis to assess differences between IVT and MFO.

A 2 x 3 (exercise intensity x obesity classes) factorial ANCOVA with repeated measures on exercise intensity and age as covariate was conducted to assess both main effects and interactions. Moreover, for HR, %HR_max_,VO_2_, and %VO_2_ the absolute variation (Δ = value at IVT—value at MFO) were calculated for all subjects. A One-way ANCOVA was conducted to determine a statistically significant difference among obesity classes on ΔHR, Δ%HR_max_, ΔVO_2_, and Δ%VO_2_ controlling for age, followed by post hoc analysis (Bonferroni adjustment). A One-way ANCOVA was conducted to determine a statistically significant difference between obesity classes on MFO, and VO_2_ (ml/min), controlling for age, followed by post hoc analysis (Bonferroni adjustment).

All statistical analyses were performed with the SPSS statistical package (Version 23.0 for Windows; SPSS Inc., Chicago, IL, USA). Statistical significance was set at P ≤ 0.05.

## Results

Subjects’ anthropometric and physiological characteristics are depicted in Table 1. Regarding the anthropometric characteristics, significant difference for weight was found (OBI vs OBII (P<0.001); OBI vs and OBIII (P<0.001); OBII vs OBIII (P<0.05) among groups (Table 1). No statistical differences were found between pre-menopause and menopause condition for HR (P = 0.06), %HR (P = 0.72), VO_2_ (P = 0.88), %VO2 (P = 0.35) at IVT and for HR (P = 0.10), %HR (P = 0.67), VO_2_ (P = 0.92), %VO2 (P = 0.22) at MFO.

**Table 1:** Anthropometrics and physiological characteristics of women in obesity class I (OBI), obesity class II (OBII) and obesity class III (OBIII).

	OBI (N = 16)	OBII (N = 20)	OBIII (N = 16)
Age (years)	42.0 ± 11.0	44.0 ± 11.0	45.0 ± 11.0
Height (m)	1.59 ± 0.0	1.62 ± 0.0	1.59 ± 0.0
Weight (kg)	83.6 ± 8.7	101.5 ± 9.6**	113.0 ± 14.4**^,^*
BMI (kg/m^2^)	32.7 ± 1.5	38.3 ± 1.3**	44.4 ± 3.8**^,^^a^
%FM (%)	41.7 ± 2.8	46.6 ± 3.8^b^	48.9 ± 5.8**
FFM (kg)	48.6 ± 5.0	54.1 ± 6.4^b^	57.2 ± 7.2**
VO_2peak_ (ml/kg/min)	23.1 ± 2.6	20.9 ± 2.9^b^	19.4 ± 1.9**
VO_2peak_ (ml/min)	1930.5 ± 251.5	2116.7 ± 338.5	2199.4 ± 346.5^b^
VO_2peak_ (ml/min/FFM)	39.7 ± 4.3	39.2 ± 5.4	38.4 ± 3.7
MET_peak_	6.6 ± 0.7	5.9 ± 0.8^b^	5.5 ± 0.5**
HR_peak_ (bpm)	150.6 ± 16.6	148.7 ± 15.2	147.1 ± 21.4
% HR_max_ (%)	84.2 ± 8.0	83.8 ± 6.6	83.2 ± 9.9

BMI = Body mass index; %FM = percentage of Fat Mass; FFM = Fat Free Mass in kilograms, VO_2peak_ = peak oxygen uptake expressed in ml/kg/min, VO_2peak_ = peak oxygen uptake expressed in ml/min, MET = Metabolic equivalent (MET = VO_2_ expressed as ml/kg/min divided 3.5), HR_peak_ = peak of heart rate, %HR_max_ = percentage of maximal heart rate. Values are mean ± SD.

*P<0.05 vs OBII;

**P<0.001 vs OBI;

^a^ P<0.01 vs OBII;

^b^ P<0.05 vs OBI.

Percentage of fat mass and fat free mass was significantly lower in OBI than the others two obesity classes (Table 1). Moreover, fat free mass was significantly lower in OBI than OBII (P<0.05) and OBIII (P<0.001) (Table 1). VO_2peak_ expressed as ml/kg/min was significantly higher in OBI than the others two obesity classes (OBI vs OBII, P<0.05; OBI vs OBIII, P<0.05). Indeed, when VO_2_ is expressed as ml/min was significantly higher in OBIII than OBI (P<0.05).

Results of the Pearson correlation indicated that for each HR (R = 0.94, P<0.01), %HR_max_ (R = 0.93, P<0.01), VO_2_ (R = 0.82, P<0.01) and %VO_2peak_ (R = 0.76, P<0.01) variable there was a significant positive correlation between IVT and MFO.

A significant main effect of exercise intensity on %VO_2peak_ controlling for age was observed (F_1,48_ = 5.2, p = 0.03). Significant exercise intensity x obesity class interactions on VO_2_ (F_2,48_ = 3.9, p = 0.03), and %VO_2peak_ (F_2,48_ = 5.1, p = 0.01) controlling for age were observed. Moreover, significant main effects of obesity class on ΔVO_2_ (F_2,48_ = 3.9, p = 0.02)_,_ and Δ%VO_2peak_ (F_2,48_ = 5.1, p = 0.01) controlling for age were observed.

Post-hoc analysis showed that %VO_2peak_ was lower at MFO than IVT (73.0±11.0% and 70.2±12.7% at MFO and IVT, respectively). Moreover: ΔVO_2_ was significantly higher (p = 0.02) in OBII than OBIII (1.0±1.5 and -0.3±1.5 ml/min/kg in OBII and OBIII, respectively), and Δ for %VO_2peak_ was significantly higher (p = 0.01) in OBII than OBIII (6.6±9.4 and -1.7±7.6% in OBII and OBIII, respectively). Physiological results at IVT and MFO are showed in Table 2.

**Table 2:** Physiological results at IVT and MFO in obesity class I (OBI), obesity class II (OBII) and obesity class III (OBIII).

	OBI(N = 16)		OBII(N = 20)		OBIII(N = 16)	
Variables	IVT	MFO	IVT	MFO	IVT	MFO
VO_2_ (ml/kg/min)	15.9 ± 1.9	15.3 ± 1.9	14.9 ± 2.5	13.9 ± 3.1	14.6 ± 2.1	15.0 ± 2.1
VO_2_ (ml/min)	1340.6 ± 228.5	1287.7 ± 236.7	1504.8 ± 232.3	1398.5 ± 273.5	1659.8 ± 322.9**	1694.0 ± 305.5^a^
%VO_2peak_ (%)	69.2 ± 8.1	66.8 ± 9.6	73.7 ± 12.9	67.2 ± 14.6	75.7 ± 10.2	77.3 ± 10.1
HR (bpm)	120.7 ± 14.4	118.2 ± 15.4	121.7 ± 15.5	118.5 ± 17.3	127.5 ± 18.1	128.1 ± 19.3
%HR_max_ (%)	67.6 ± 8.7	68.7 ± 8.8	72.1 ± 8.2	66.2 ± 9.1	66.9 ± 9.7	72.5 ± 9.3
Slope (%)	0.9 ± 1.8	0.5 ± 1.6	0.4 ± 1.1	0.4 ± 1.4	0.3 ± 1.5	0.5 ± 1.6
GS (km/h)	4.8 ± 0.6	4.6 ± 0.7	4.5 ± 0.8	4.2 ± 1.0	4.2 ± 0.7	4.1 ± 0.8
RPE	4.3 ± 1.8	3.8 ± 1.6	4.8 ± 2.1	3.4 ± 1.9	4.2 ± 1.6	4.1 ± 1.7

VO_2_ = oxygen uptake expressed in ml/kg/min; %VO_2peak_ = percentage of VO_2peak_; HR = Heart rate; GS = gait speed; RPE = Rate of Perceived Exertion,

**P<0.01 vs OBI;

^a^P<0.01 vs OBI and OBII

**Fig 1:**
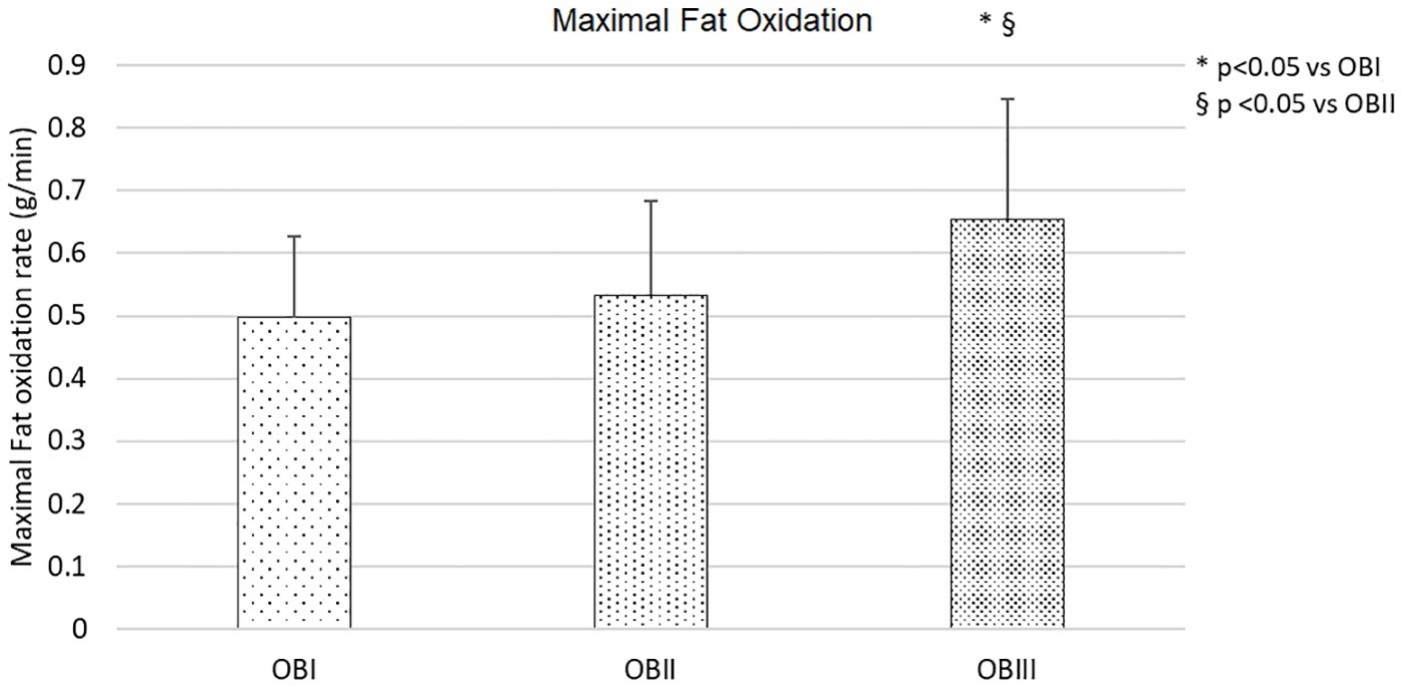
Maximal fat oxidation rates expressed in g/min in obesity class I (OBI), obesity class II (OBII) and obesity class III (OBIII).

The rates of maximal fat oxidation are presented in Fig 1. Maximal Fat Oxidation rates was significantly higher (p<0.05) in OBIII (0.65 ± 0.18 g/min) than the other two obesity classes (OBI = 0.50 ± 0.13 g/min and OBII 0.53 ± 0.15 g/min) (Fig 1).

VO_2_ expressed as ml/min was significantly higher in OBIII subjects than subjects of the other two classes at both MFO (P<0.01) and at IVT.

The “low” and “high” borders of Fat_max_ for subjects of each obesity group are depicted in Table 3. Significant differences were found between OBIII and OBI in low and high borders for gait speed (p<0.05). In all subjects the exercise intensities corresponding to IVT was within the Fat_max_ zone.

**Table 3:** Low and high borders of Fat_max_ (fat max zone) in obesity class I (OBI), obesity class II (OBII) and obesity class III (OBIII).

	OBI (N = 16)		OBII (N = 20)		OBIII (N = 16)	
Variables	Low	High	Low	High	Low	High
VO_2_ (ml/kg/min)	12.7 ± 2.0	18.2 ± 2.0	12.0 ± 2.4	17.4 ± 4.5	12.9 ± 1.7	16.7 ± 2.7
HR (bpm)	109.2 ± 14.6	129.9 ± 16.1	111.1 ± 13.9	129.8 ± 19.0	118.7 ± 15.1	136.1 ± 20.0
GS (km/h)	3.7 ± 0.7	5.2 ± 0.5	3.4 ± 0.8	4.9 ± 0.6	3.2* ± 0.7	4.7* ± 0.6
RPE	2.4 ± 1.0	5.6 ± 2.4	2.9 ± 1.7	5.5 ± 1.8	2.9 ± 1.2	5.5 ± 1.8

VO_2_ = oxygen uptake expressed in ml/kg/min, HR = Heart rate; GS = gait speed; RPE = Rate of Perceived Exertion;

*P<0.05 vs OBI

## Discussion

The main objective of this study was to determine the relationship between IVT and MFO in women with class I, II and III obesity. It has been known that the use of IVT as a parameter to individualize exercise intensity might be helpful to improve body composition and physical fitness in obese adults [4, 6]. Moreover, the intensities at which the MFO occurs (Fat_max_) has been used for the prescription of PA, especially in weight loss programs, to decrease body mass, BMI and, percentage of body fat [13, 28]. However, different obesity conditions may influence not only physical performance but also substrate utilization during moderate exercise intensity. The results presented in this manuscript demonstrate that the use of HR and VO_2_ corresponding to IVT could be a useful parameter to prescribe PA with the aim of improving cardio-respiratory efficiency and maximizing fat oxidation in obese subjects. Moreover, our results showed that MFO was higher in OBII than the other two obesity classes, and this difference might be justified by higher VO_2_ expressed as ml/min in OBIII group than OBI and OBII groups.

As we expected, obesity groups differ among them in regards of weight and BMI and %FM and FFM were significantly lower in OBI subjects than the other two obesity classes. There were no significant differences in anthropometrics characteristics between OBII and OBIII subjects. Our results showed that subjects in the OBI group had higher peak oxygen consumption as expressed in ml/kg/min compared to the other groups. Conversely, when oxygen consumption is expressed as ml/min, VO_2_ was higher in OBIII than in OBI. This result might be explained by the significant higher FFM in OBIII than OBI. Moreover, when VO_2_ is expressed as ml/min/FFM no significant differences were observed between groups. These results indicate that lower body weight allows to reach higher expression of physical efficiency but do not influence cardiorespiratory fitness.

As expected, VO_2peak_ values were very low in all obesity groups as previously demonstrated by our group [6, 7]. In fact, during sub-maximal incremental test, changes are observable in physiologic parameters that are altered in obese subjects as the inability to augment ventilation and the ability to manage the increased oxygen request during the exercise; these alterations are also due to the biomechanical issues correlated to the movement of the obese subjects [29].

Even if cycle ergometer has been more often used for CPET tests in obese subjects, due to the lower load on joints than a treadmill, we choose the latter because the walking speed, the HR and the RPE corresponding to IVT might be used in order to lead the subjects to benefit from regular un-supervised physical activity in an almost daily routine as, for instance, regular walking.

Our results showed that there were not significant differences on HR and VO_2_ between IVT and MFO, indicating that prescribing exercise using IVT might also optimize fat utilization. Substrate utilization is commonly expressed as VO_2_ and %VO_2peak_, however, these parameters do not directly translate to exercise prescription. On the other hand, heart rate is largely used in the prescription of PA [6, 30]. However, since HR might have an high inter-variability between subjects it could be better to use the heart rate plus other variables such as gate speed and RPE in order to prescribe exercise intensity in obese subjects.

Dasilva et al. [16] demonstrated that, when walking on a treadmill, healthy adults tend to self-select exercise intensities that fall within the Fat_max_ zone which we showed to include the intensity corresponding to IVT. Bircher et al. [14] demonstrated that, compared to athletes, obese adults reach the MFO at lower intensities (65%VO_2peak_) and their substrate utilization starts shifting from fat to carbohydrate at lower exercise intensities. These results agree with previous results published from our and other groups that depicted IVT at low exercise intensities in obese individuals [7, 31] supporting the hypothesis that obese subjects prefer low intensities activities allowing them to better adhere to PA [32]. Moreover, low walking speeds and HR has been shown to improve cardiorespiratory fitness in obese subjects highlighting the dual benefit of exercising at intensities corresponding to IVT [7].

The significant exercise intensity x obesity classes difference for VO_2_, and %VO_2_ indicates that the relationship between IVT and MFO might be different among obesity classes. Indeed, the results of ΔHR, Δ%HR, ΔVO_2_, and Δ%VO_2peak_ indicated that OBIII had a lower value than OBII. In fact, in OBI and OBII, HR and VO_2_ were slightly lower at MFO than IVT, on the contrary in OBIII, HR and VO_2_ were slightly higher at MFO than IVT.

We also analyzed maximal fat oxidation and determined the Fat_max_ zone, considering ±10% from MFO (Table 3). In regards of the results of Fat_max_, we might affirm that IVT was always in the Fat_max_ zone, and that subjects belonging to OBIII had a higher fat oxidation than subjects in the other groups. In fact, in OBIII subjects the high rates of MFO obtained during the incremental test, showed that subjects having a BMI≥40 kg/m^2^ had a higher fat oxidation than the other groups. This result may be justified by the higher %FM in OBIII. Speed at low and high borders of Fat_max_ zone differ between OBIII and OBI. In detail, the OBI group presented high value of speed in the Fat_max_ zone than OBIII. Indeed, different parameters, such as training and acute nutritional status of the subjects population and/or the exercise modality (running or cycling) might influence MFO and Fat_max_ results [33]. Our MFO results were higher than those reported by other studies in obese subjects (MFO ranging from 0.25 to 0.4 g/min) [9,34,35,36]. These differences might by justify by the different graded exercise protocol, and acute nutritional status of the subjects during the experimental test. In details, scientific evidence [37] showed that fat oxidation rates were 28% higher when walking compared with cycling in moderately trained male subjects. Bordenave et al. [38] showed that using a 3-min step protocol an average overestimation of MFO occurred. Moreover, Ara et al. [34] showed that in obese subjects, MFO during exercise was increased compared to normal body weight subjects. In our study, subjects were obese and they perform an incremental exercise test on treadmill at least one hour and half after breakfast. Moreover it could be possible that subjects in our study had a high-fat breakfasts. Therefore, these conditions might influence the MFO in our subjects resulting in a higher MFO than those reported by other studies. Moreover, the high standard deviation in our study cohort might be due to other variables that might influences the fat oxidation rate (e.i. menstrual cycle, breakfast food) that we did not assess.

Our study suggests that individuals in the third class of obesity need to be treated specifically when prescribing PA considering that MFO and exercise intensity at which MFO occurs were higher, and that the gait speed corresponding to the low and high border of Fat_max_ zone were lower in OBIII compared to the other two obesity classes.

Scientific evidences showed that training at Fat_max_ exercise intensity has positive effects on body composition and cardiorespiratory fitness. In details, Tan et al. [39] have demonstrated that Fat_max_ training enhances body fat mass loss and improves VO_2max_. Moreover Drapier et al. [28] showed that low intensity exercise training (at Fat_max_) maintains its weight-reducing effect 3 years, while diet, used as a comparator, is no longer efficient. Moreover, exercise intensity corresponding to Fat_max_ leads the exercise to be better tolerated and therefore it could be helpfully to allow obese individuals to get used to exercise before progressing.

This result highlights the possibility to use the Fat_max_ exercise intensity also for daily routine training.

IVT training is defined as an effective method to enhance physical efficiency, especially in obese subjects [4, 6]. Our results showed that differences between IVT and MFO are not relevant in the prescription of PA in obese adult women. Therefore, it is important to identify IVT in individuals with different degrees of obesity to optimize MFO. Makni et al. [40] demonstrated that in obese children, the 6MWT can be used to predict Fat_max_. Moreover, our group has previously [22] demonstrated that using a 6MWT it might be possible to estimate the exercise intensity expressed as HR corresponding to the IVT in obese adults. Therefore, this equation may be useful also to prescribe exercise intensity at which MFO occurs in women with obesity. However, future studies are needed in order to directly assess the relationship between 6MWT and MFO in obese individuals.

In conclusion, the results of this study demonstrate that the differences between IVT and MFO are negligible since they are not relevant for practical application in obese women. However, for the practical applications it is recommended to take into consideration the high HR inter-variability between subjects.

However, obese subjects with a BMI≥40 showed to have a higher MFO compared to obese subjects with lower BMI. The HR, %HR and VO_2_, corresponding to IVT and MFO are not different between subjects with different BMI. Therefore, prescribing exercise intensity at IVT might be useful not only to increase cardiorespiratory efficiency but also to maximize fat oxidation in women with class I, II and III obesity.

### Study limitations

We are aware that we assessed the VO_2peak_ and not the VO_2max_ since the graded exercise test was interrupted when subjects declared to perceive a fatigue corresponding to a value of 10 on RPE-OMNI-Walk/Run Scale. This choice might influence the data regarding the percentage of the maximum (%VO_2max_) at which IVT and MFO occur. However, due to the subjects’ characteristics we did not assessed the subjects’ VO_2max_. We chose to analyze VO_2peak_ for two reasons: first, physical and physiological characteristics typical of obese subjects might not lead to respect all the criteria needed to assess VO_2peak_; secondly, the primary aim of this study was to compare the exercise intensity corresponding to IVT and MFO (moderate exercise intensity) and not the maximal exercise capacity in obese subjects. Nevertheless, future studies are needed to assess the relationship between IVT and MFO in both genders and in non-obesity condition. Regarding the MFO evaluation, we know that there are different parameters, such as training and acute nutritional status, menstrual cycle, and graded exercise protocol that might influence the results and that we did not assessed subjects breakfast characteristics. However, the primary aim of this study was not to assess the MFO with a standard protocol (5-min, 35-W step increments performed after an overnight fast) or to assess the differences in MFO among obesity classes, but to investigate if there were differences on exercise intensity between MFO and IVT in women with obesity.

## Supporting information

S1 File Supporting information file. Cond = menopause (1 = yes, 0 = no); Cla = classes; HR_max_ = 220-age; HR_rip_ = heart rate at rest; BMI = body mass index; pFM = percent of fat mass; FFM = fat free mass; VO_2p_ = oxygen uptake at peak expressed as ml/min/kg; aVO_2p_ = oxygen uptake at peak expressed as ml/min; VO2FFM = oxygen uptake at peak expressed as ml/min/FFM; MET_p_ = metabolic equivalent at peak; HR_p_ = heart rate at peak; pHR_max_ = heart rate at peak expressed as percentage of heart rate maximum. HR_ivt_ = heart rate at individual ventilatory threshold. HRMFO = heart rate at maximal fat oxidation; ΔHR = hr at IVT—HR at MFO; pHR_maxivt_ = heart rate at individual ventilatory threshold expressed as percentage of heart rate maximum; pHR_maxMFO_ = heart rate at maximal fat oxidation expressed as percentage of heart rate maximum; ΔpHR_max_ = pHR_maxivt_–pHR_maxMFO_; VO_2ivt_ = oxygen uptake (ml/min/kg) at individual ventilatory threshold; VO_2MFO_ = oxygen uptake (ml/min/kg) at maximal fat oxidation; ΔVO_2_ = VO_2ivt_-VO_2MFO_; aVO_2ivt_ = oxygen uptake (ml/min) at IVT; aVO_2MFO_ = oxygen uptake (ml/min) at maximal fat oxidation; pVO_2peakivt_ = oxygen uptake at individual ventilatory threshold expressed as percentage of oxygen uptake at peak; pVO_2peakMFO_ = oxygen uptake at maximal fat oxidation expressed as percentage of oxygen uptake at peak; ΔpVO_2_ = pVO_2peakivt_-pVO_2peakMFO_; SP = speed; SL = slope; SL_ivt_ = slope at individual ventilatory threshold; RPE_ivt_ = rate of perceived exertion at individual ventilatory threshold; RPE_MFO_ = rate of perceived exertion at maximal fat oxidation; MFO = maximal fat oxidation; HRL = heart rate at low border of fat max zone; HRH = heart rate at high border of fat max zone; VO_2_L = oxygen uptake at low border of fat max zone; VO_2_H = oxygen uptake at high border of fat max zone; Fat_max_L = fat oxidation at low border of fat max zone; Fat_max_H = fat oxidation at high border of fat max zone; RPEL = rate of perceived exertion at low border of fat max zone; RPEH = rate of perceived exertion at high border of fat max zone; SL = speed at low border of fat max zone; SH = speed at high border of fat max zone;
(XLSX)
